# Antimicrobial and Antibiofilm Properties of Hydroxyapatite/Nano-Hydroxyapatite in Preventing Dental Caries: A Systematic Review

**DOI:** 10.1055/s-0045-1802568

**Published:** 2025-05-01

**Authors:** Nurdiana Dewi, Meirina Gartika, Dwi Gustiono, Dikdik Kurnia, Arief Cahyanto

**Affiliations:** 1 Doctoral Program, Faculty of Dentistry, Universitas Padjadjaran, Bandung, Jawa Barat, Indonesia; 2 Department of Pediatric Dentistry, Faculty of Dentistry, Universitas Lambung Mangkurat, Banjarmasin, Kalimantan Selatan, Indonesia; 3 Department of Pediatric Dentistry, Faculty of Dentistry, Universitas Padjadjaran, Bandung, Jawa Barat, Indonesia; 4 Research Center for Advanced Materials, National Research and Innovation Agency, Tangerang Selatan, Banten, Indonesia; 5 Department of Chemistry, Faculty of Mathematics and Natural Science, Universitas Padjadjaran, Sumedang, Jawa Barat, Indonesia; 6 Department of Clinical Sciences, College of Dentistry, Ajman University, Ajman, United Arab Emirates; 7 Centre of Medical and Bio-allied Health Sciences Research, Ajman University, Ajman, United Arab Emirates

**Keywords:** biofilms, caries, hydroxyapatites, microbiota, systematic review

## Abstract

Controlling biofilm is a crucial strategy and an essential component of preventing dental caries. Considerable research has been conducted in recent years on the clinical application of hydroxyapatite (HAp) and hydroxyapatite nanoparticles (nHAp) in preventing dental caries. However, these studies have yet to investigate the effectiveness or mechanism of these substances as antibacterial and antibiofilm agents. This study aimed to provide a thorough analysis of the current evidence on the antibacterial and antibiofilm characteristics of HAp/nHAp in the prevention of dental caries. Searches were conducted across five databases: Cochrane Library, PubMed, Scopus, EBSCOhost, and ScienceDirect. Google Scholar was also searched. Titles, abstracts, and full text were evaluated following the guidelines set by the Preferred Reporting Item for Systematic Review and Meta-Analyses (PRISMA). A methodological quality assessment of the studies was conducted using the QUIN tool. The initial retrieval totaled 15,047 studies, from which 3,487 were excluded. A total of 11,560 studies were screened based on the title and abstract, resulting in 24 full-text studies considered potentially eligible for inclusion ( *κ* = 0.9599). Finally, 19 studies met all the defined inclusion criteria and were included in this comprehensive systematic review ( *κ* = 0.8837). HAp/nHAp demonstrates antimicrobial activities against gram-negative and gram-positive bacteria and fungi. However, nHAp's antibiofilm efficacy remains limited. Further investigation is required to improve the efficacy of antibacterial and antibiofilm agents.

## Introduction

In the oral cavity, microorganisms can live either freely (planktonic) or as a component of a biofilm. These organisms exhibit unique traits that facilitate their strong adherence to tooth surfaces, generate greater quantities of acid from fermentable sugars compared with other oral bacterial species, and demonstrate superior survival in acidic environments. Biofilms have distinct properties compared with planktonic microorganisms. Biofilms are structured ensembles of microorganisms that consist of extracellular polymeric substances (EPS) synthesized by the organisms. 1 It is defined by alterations in the permanent attachment of microbial cells to surfaces or to one another, encapsulated in EPS, and displaying distinct phenotypes for gene expression and growth rates. A biofilm consists of either a single microorganism or a mixture of bacteria, fungi, archaea, protozoa, and yeasts. Biofilms exhibit greater resistance to bactericidal antimicrobials compared with planktonic cells. 2 3 Cariogenic biofilms arise and expand in the presence of a diet abundant in fermentable carbohydrates. Lactic acids and various other organic acids are generated as by-products of glucose metabolism and permeate from the biofilm. 4

Dental caries is a significant global health issue, with *Streptococcus mutans* identified as a principal pathogen of this disease. 5 Subsequent research, however, demonstrated that caries can occur without the presence of *S. mutans* . This organism may endure on other intact surfaces, so it has been established that *S. mutans* alone cannot serve as a predictor of carious risk. Recent genetic study has revealed other microorganisms that may contribute to caries formation. 6 Numerous other organisms have been obtained from carious sites or identified as distinctly present during the caries development process, and they have been broadly suggested to be associated with caries. 7 These microbes comprise gram-positive cocci ( *S. mutans* , *S. sobrinus* , *S. mitis* , *S. salivarius* , *S. sanguinis* , *S. intermedius* , *S. vestibularis* , *Staphylococcus aureus* , *Atopobium* spp., *Peptostreptococcus* spp., *Enterococcus fecalis* ), gram-positive rods ( *Actinomyces odontolyticus* , *A. naeslundii* , *A. viscosus* , *A. israelii* , *Limosilactobacillus fermentum* , *Lactobacillus acidophilus* , *Bifidobacterium dentium* , *Propionibacterium* spp.), gram-negative cocci ( *Veillonella parvula* , *Neisseria* spp.), gram-negative rods ( *Treponema denticola* , *Bacteroides melaninogenicus* , *Fusobacterium necrophorum* , *F. mortiferum* , *Escherichia coli* , *Klebsiella pneumoniae* , *Enterobacter aerogenes* , *Citrobacter freundi* , *Pseudomonas fluorescence* , *Haemophilus* spp., *Prevotella* spp., *Leptotrichia* spp.), and fungi ( *Candida albicans* , *C. tropicalis* , *C. glabrata* ). 6 8 *Streptococcus salivarius* is a commensal bacterium prevalent in the oral cavity of healthy individuals. Certain strains have demonstrated anti-inflammatory activities, synthesize bacteriocins, and function as antagonists to various bacterial species, including those with cariogenic potential. 9

Biofilm control is one of the most essential strategies and fundamental elements of the preventive management of dental caries. Mechanical brushing to disrupt the biofilm's structure is a highly effective method for disease management. 10 Active compounds are commonly utilized in conjunction with mechanical biofilm control. Antimicrobial agents can potentially impede the proliferation of cariogenic bacteria and reestablish the biological balance within the oral ecosystem. 11 The application of nanotechnologies, particularly hydroxyapatite (HAp), chitosan, metals, and metal oxide nanoparticles, has drawn the attention of researchers searching for novel bioactive compounds to stop the onset and progression of dental caries. 12 13 One alternative is inorganic nanoparticles, which have a far lower probability of inducing bacterial resistance than small-molecule antibiotics. This is because nanoparticles frequently use a variety of mechanisms to carry out their antimicrobial effects, which hinder bacteria's ability to adapt and develop resistance. 14

The mineral calcium apatite, known as HAp, has the molecular formula Ca _10_ (PO _4_ ) _6_ (OH) _2_ . It has a calcium-to-phosphorus molar ratio of 1.67. It is comparable in chemical composition to the apatite crystal in human bone and enamel. HAp possesses a crystal lattice structure. 15 Nano-hydroxyapatite (nHAp) is a nanomaterial with very small crystal grain sizes. 16 Nanoparticles often exhibit atypical physical and chemical characteristics due to their small dimensions. 17 nHAp has more solubility, surface energy, and bioactivity than HAp, and its structure closely resembles that of dental apatite. 18 19

The clinical application of HAp and nHAp in preventing dental caries has been extensively investigated in recent years. However, none have addressed the substance's effectiveness or mechanism as an antibacterial and antibiofilm agent. The effectiveness of nHAp against bacteria and biofilms implicated in the development of caries has been documented in a published systematic review; however, HAp was mixed with other substituted ions. 20 This systematic review intended to comprehensively analyze the literature to critically evaluate and delineate the evidence on the antibacterial and antibiofilm characteristics of HAP/nHAp in the prevention of dental caries.

## Methods

### Review Design

The study protocol was registered with Open Science Framework (OSF), and a registration DOI was assigned at https://doi.org/10.17605/OSF.IO/CDJXP or https://osf.io/cdjxp/ . Throughout the current systematic review, the Preferred Reporting Items for Systematic Reviews and Meta-Analyses (PRISMA) were used (the completed PRISMA-S checklist can be found in the Supplementary Materials S1 and S2 , available in the online version only). 21 The formulation of the review question and selection of the suitable research instrument was derived from the PICOS paradigm, an acronym for participant, intervention, comparison, outcome, and study. 22 The independent variable was hydroxyapatite/nano-hydroxyapatite (HAp/nHAp), and the primary outcome measure was antimicrobial or antibiofilm properties in preventing dental caries.

### Search Strategy

A comprehensive database search was conducted using well-known electronic databases such as Cochrane Library, PubMed, Scopus, EBSCOhost, ScienceDirect, and Google Scholar. The literature database was queried using MeSH terms, keywords, and other relevant phrases associated with nHAp, microbiota, biofilm, and dental caries. The Boolean operators “AND” and “OR” combined both terms as shown in Table 1 . Furthermore, a comprehensive search was conducted by analyzing the reference lists of pertinent research and manually searching for other potentially suitable publications, including literature published until November 2024.

**Table 1:** Search strategy in different databases

Database	Search strategy
Cochrane	((hydroxyapatite[MeSH Terms]) AND (((microbiota[MeSH Terms]) OR (bacteria[MeSH Terms])) OR (biofilm[MeSH Terms]))) explode all trees) OR (MeSH descriptor: [Biofilms] explode all trees)) OR (MeSH descriptor: [Dental Caries] explode all trees)
PubMed	((hydroxyapatite[MeSH Terms]) AND (((microbiota[MeSH Terms]) OR (bacteria[MeSH Terms])) OR (biofilm[MeSH Terms]))) AND (dental caries[MeSH Terms])
Scopus	(TITLE-ABS-KEY (hydroxyapatite)) AND ((TITLE-ABS-KEY (microbiota)) OR (TITLE-ABS-KEY (bacteria)) OR (TITLE-ABS-KEY (biofilm))) AND (TITLE-ABS-KEY (“dental caries”))
EBSCOhost	(hydroxyapatite) AND (microbiota OR bacteria OR biofilm) AND (“dental caries”)
ScienceDirect	(hydroxyapatite) AND (microbiota OR bacteria OR biofilm) AND (“dental caries”)
Google Scholar	(hydroxyapatite) AND (microbiota OR bacteria OR biofilm) AND (“dental caries”)

### Eligibility Criteria and Study Selection Process

The PICOS model was employed to structure the clinical research question by defining the inclusion and exclusion criteria ( Table 2 ). The inclusion criteria were studies written in English, conducted *in vitro* , pertaining to the antimicrobial or antibiofilm effects of HAp/nHAp on organisms associated with caries, and containing only HAp as an active ingredient. Abstracts without the corresponding full papers, studies published in case reports, book chapters, conference proceedings, patents, editorial letters, literature reviews, systematic reviews, and meta-analysis papers were excluded from consideration. No defined limitations were set on the potential outcomes of the control treatment, which may encompass a placebo, untreated control, or standard control such as fluoride or chlorhexidine. Additionally, no restrictions concerning the outcome were defined.

**Table 2:** The PICOS framework

P	Participant: microbial or biofilm that plays a role in dental caries based on Yadav and Prakash 8 and Zhang et al 6
I	Intervention: hydroxyapatite/nano-hydroxyapatite (HAp/nHAp)
C	Comparison: alternative treatments such as placebo, untreated control, or conventional control (such as fluoride or chlorhexidine)
O	Outcome: antimicrobial or antibiofilm activities of hydroxyapatite/nano-hydroxyapatite (HAp/nHAp)
S	Studies: *in vitro* study

Two reviewers (N.D. and M.G.) independently examined the titles and abstracts of the retrieved papers for relevance and agreement with the study goals. The reviewers were not provided with any information that would conceal the names of the journals or authors of the articles, their affiliations, or the findings of their studies. A third reviewer (D.G.) was consulted and discussed to reach a consensus regarding study inclusion and data extraction. The complete texts of the chosen articles were carefully examined following the initial screening. Cohen's kappa was used to determine the inter-rater reliability.

### Data Synthesis

A comprehensive qualitative analysis was performed on the studies that met the inclusion criteria. One reviewer (N.D.) initially extracted data from the eligible studies, which was subsequently validated by a second reviewer (M.G.). Disagreements were deliberated and resolved by careful examination of the source, leading to the establishment of a consensus. The data collected in predefined Microsoft Excel spreadsheets, including the author's name, year of publication, experimental group, concentration, source of HAp/nHAp product, particle size, microbial species, study design, outcome measured, and study conclusion, were systematically tabulated. Duplicates were manually removed once the studies were listed alphabetically. The completed screener and extractor instructions can be found in Supplementary Materials S3 and S4 (available in the online version only). The inter-rater reliability was calculated using Cohen's kappa.

### Risk of Bias Assessment

The full papers that met the eligibility criteria were objectively analyzed by two reviewers, N.D. and M.G., who meticulously assessed them for methodological risk of bias. The papers were evaluated for methodological quality using the Quality Assessment Tool for *In Vitro* papers (QUIN tool) standards. These guidelines include 12 criteria for reporting randomized clinical trials for *in vitro* studies. 23 24 The assessment was conducted to evaluate the chosen study's quality and possible biases.

## Results

An initial search yielded 15,047 studies, out of which 3,487 were eliminated due to the presence of case reports, book chapters, conference proceedings, patents, editorial letters, literature reviews, systematic reviews, and meta-analysis papers. Out of the 11,560 studies, 11,536 were eliminated after evaluating their titles and abstracts, resulting in 24 full-text studies that were considered potentially eligible for inclusion (inter-rater reliability, *κ* = 0.9599). Five studies were eliminated due to the presence of other active components apart from HAp, 25 26 27 28 and the study did not analyze the antibacterial and antibiofilm capabilities of HAp/nHAp against caries-causing microbes. 29 Ultimately, 19 studies satisfied all the criteria for inclusion and were included in this systematic review (inter-rater reliability, *κ* = 0.8837).

These studies showed substantial variation in terms of methodology, materials, microorganisms, and outcomes. Among the reviewed studies, 16 examined the effects of nHAp on bacteria and/or single-species biofilms. One study explored the effects of nHAp on single- and multispecies biofilms, and one study investigated the effects of nHAp on bacteria and cross-kingdom biofilms. The single pathogen bacterial species examined in this systematic review were *S. mutans* , *S. sobrinus* , *S. gordonii* , *S. aureus* , *E. coli* , and *E. faecalis* . The single fungal species was *C. albicans* and the single nonpathogen bacterial species was *S. salivarius* . The multibacterial species used in the studies of this systematic review were *S. mutans* and *S. gordonii* . The cross-kingdom species used in the studies of this systematic review were *S. mutans* and *C. albicans* .

The particle size range of the HAp reviewed was 2 to 5 μm for micro-HAp and 8 to 1,870 nm for nHAp. The HAp sources used are commercial, 6 30 31 32 33 34 35 36 *Atactodea glabrata* snail shells, 37 egg shells, 38 sheep bones, 38 *Jania rubens* red seaweeds, 39 *Corallina officinalis* red seaweeds, 39 snakehead ( *Channa striata* ) fish bones, 40 buffalo bones, 41 direct mixing Ca ^2+^ and PO _4_^2−^ precursor, 42 bovine bones, 43 porcine bones, 43 marble, 44 and microwaved-synthesized nHAp. 45 Two studies did not specify the source of HAp. 46 47

**Fig. 1:**
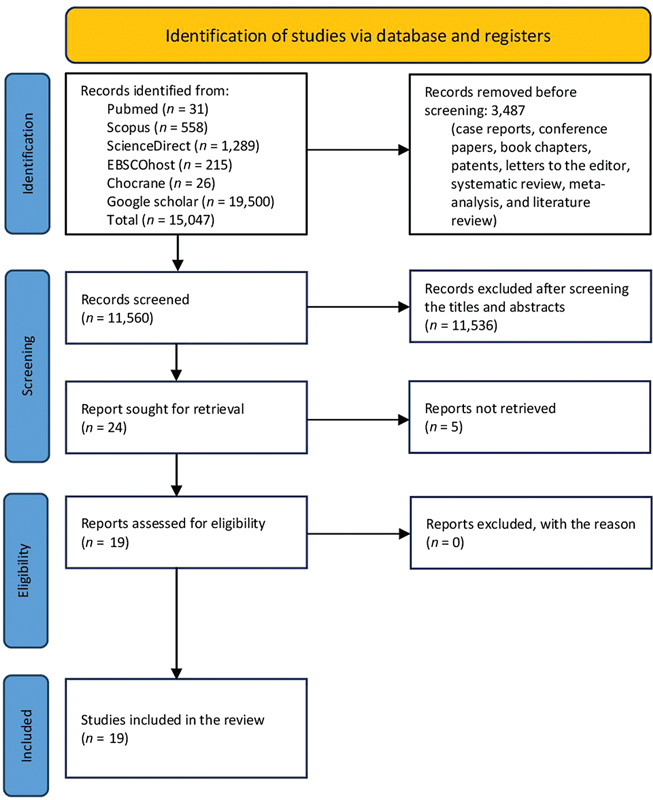
The Preferred Reporting Item for Systematic Review and Meta-Analyses (PRISMA) flow diagram illustrates the process of reviewing and selecting studies. 88

Fig. 1 presents the entire process of article collection, screening, and eligibility assessment using the strategies outlined in the “Methods” section. Table 3 displays the primary results of studies that examined the impact of HAp/nHAp on organisms and biofilms associated with caries. Fourteen studies used nHAp, and one study used both HAp and nHAp. The results of the articles reviewed were heterogeneous.

**Table 3:** Summary of the effect of hydroxyapatite/nano-hydroxyapatite (HAp/nHAp) as antimicrobial and antibiofilm

Study	Intervention	HAp/nHAp details	Study model and design	Outcome measured	Study conclusion
Luo et al 31	• 10% nHAp • 10% Disaggregated nano-hydroxyapatite (DnHAp) • 10% nHAp • 10% micro-HAp • Sterile deionized water (DDW)	• **Product** : commercially obtained from Sigma-Aldrich, America • **Particle size** : DnHAp: 167.1–666.8 nm; nHAp: 1,870 nm; micro-HAp: 2–5 μm	• **Microbial species:** single species of *Streptococcus mutans* and multispecies of *S. mutans, S. gordonii* • **Design:** four treatment groups were introduced onto a new 24-well plate, followed by the addition of samples containing biofilms into the plate containing the treatment solution. The samples were incubated with the treatment solution for 30 min. All samples were newly prepared for further testing and analysis	Biofilm metabolic activity using MTT assay and lactic acid production by lactic acid production assay	• DnHAp has the ability to hinder the metabolic activity and acid production of oral bacterial biofilm; however, it does not exert a significant influence on the composition of multispecies biofilm • Both nHAp and micro-HAp did not cause significant changes (increase/decrease) in metabolic activity and lactic acid production of single-species biofilms • nHAp greatly increased the metabolic activity and lactic acid generation of the multispecies biofilm
Huang et al 30	• 1% DnHAp • 1% nHAp • 0.33% sodium hexametaphosphate (SHMP) • Sterile deionized water (DDW)	• **Product:** commercially obtained from Sigma-Aldrich, America • **Particle size:** DnHAp: 179.4 nm; nHAp: 665.7 nm	• **Microbial species:** single species of *S. mutans* and dual species of *S. mutans* and *Candida albicans* • **Design:** the biofilms were treated with four experimental groups for 30 min and then transferred to a new plate with a fresh medium. This procedure was repeated every 12 h until the 36th h. After 48 h, the biofilm samples were harvested for further analysis	Metabolic activity analysis using MTT assay, lactic acid production measurement using lactic acid production assay, biomass biofilm, and EPS production using CLSM	• DnHAp demonstrated inhibitory effects on the metabolic activity and lactic acid production of biofilms without affecting the variety of saliva-derived microcosm biofilms • nHAp did not demonstrate inhibitory effects in metabolic activity, lactic acid generation, biomass, and EPS of single-species, cross-kingdom, and saliva-derived microcosm biofilms
El-Gar et al 32	• 30 mg/mL nHAp large particle size (LPS) • 30 mg/mL nHAp small particle size (SPS) • 5% NaF	• **Product:** nHAp LPS was synthesized at the Institute of Nano-Science and Technology; HAp-SPS was ready to make powder from Nano-Stream, Egypt • **Particle size:** nHAp LPS: length, 86.64 nm and width, 17.43 nm; HAp-SPS: length, 30–40 nm and width, 8–12 nm	• **Microbial species:** *S. mutans* • **Design:** for MIC and MBC, the strain was grown in BHI broth. The nHAp powder solution was prepared and diluted in the BHI broth. The plates were incubated at 37°C for 12–18 h; *S. mutans* were inoculated into the mitis salivarius agar to detect the antiadhesive effect. Enamel blocks were thereafter placed on the agar surface and incubated in a CO _2_ incubator at 37°C for 48 h	MIC and MBC using the microtiter broth dilution method, bacterial adhesion test by viable count technique (colony forming unit [CFU]), biofilm thickness and antibiofilm action using CLSM, and surface topography using SEM	• The nHAp-LPS suspension exhibited antibacterial, antiadhesive, and antibiofilm effects against cariogenic *S. mutans* , with no significant difference with NaF varnish • nHAp-SPS had no direct antibacterial, antiadhesive, and antibiofilm action
Park et al 33	• 0.1 or 5% nHAp in both BMM and BHI media, with or without 1% sucrose • No treatment (negative control)	• **Product:** commercially obtained from Alfa Aesar • **Particle size:** 20–50 nm	• **Microbial species:** single species of *S. mutans* • **Design:** triplicate wells were filled with either BMM or BHI, each containing equal volumes of inoculum. The wells were supplemented with 0.1 or 5% nHAp (at concentrations of 0.5 and 2.5 mg/mL) and 0 or 1% sucrose. Duration of intervention, 24 h	The kinetics of growth by the kinetics program, cell survival by CFU, biofilm formation by MTT staining, transcription of *gtf* genes by RNA extraction and qRT-PCR, amounts of polysaccharides by spectrophotometer, and formation of biofilm by CFU	nHAp stimulated bacterial growth and promoted biofilm formation in both BMM and BHI media, regardless of the presence or absence of sucrose. This effect was achieved by upregulating the expression of *gtf* genes, leading to an enhanced synthesis of glycosyltransferase responsible for the synthesis of polysaccharides
Zhang et al 48	• 10% nHAp • 18.4-mM NaF • Distilled water (negative control)	• **Product:** commercially purchased from Nano-Biomaterials Industrialization, Sichuan University, China • **Particle size:** 10–20 nm in diameter and 60–80 nm in length	• **Microbial species:** *S. mutans* • **Design:** biofilm *S. mutans* were formed on the surfaces of artificially demineralized enamel. Following 2 d of biofilm formation, the model underwent a pH-cycling schedule. The specimens were treated twice daily for 5 min until day 19	Lactic acid production by lactic acid production assay, biofilm viable cell counts by CFU, and calcium contents by atomic absorption spectroscopy	• There were no significant changes in biofilm viable count and lactic acid production in the nHAp group compared with the water and NaF groups • nHAp enhanced calcium content of the biofilms eightfold higher compared with the water and NaF groups
Ahmed et al 37	• nHAp 1 mg/mL • Snail shell powder 1 mg/mL • Gentamicin 1 mg/mL (positive control against bacteria) • Amphotericin B 1 mg/mL (positive control against fungus)	• **Product:** synthesized from *Atacdodea glabrata* snail shells • **Particle size:** 15.22 nm diameters	• **Microbial species:** single species of *S. aureus, C. albicans, Escherichia coli* • **Design:** the inoculum of the standardized culture of the test organism was spread uniformly in wells, and treatments were applied. Nutrient agar plates were used for bacteria, and malt agar plates were used for fungi. The plates were incubated at 37°C for 24 h	Diameter on inhibition zone using agar well diffusion method, MIC using tube dilution technique, antibiofilm activity using crystal violet assay, morphological alteration using inverted microscope coupled with the digital camera	• nHAp exhibited higher inhibitory activity than reference compounds against all tested organism • nHAp displayed potent antibiofilm activity against *S. aureus* and moderate biofilm inhibition against *E. coli* and *C. albicans*
Mousavi et al 38	• Eggshell nHAp (nHAp 1000–7.8 μg/mL) • Sheep bone of duck nHAp (nHAp/Sb) 1000–7.8 μg/mL • Negative control: BHI • Positive control: BHI and microorganism	• **Product:** synthesized from eggshell and sheep bones • **Particle size:** 10–70 nm	• **Microbial species:** single species of *S. aureus, E. faecalis, E. coli, Candida* • **Design:** BHI or liquid medium was filled at 96 wells, then compounds were added to the culture medium at wells, and microorganisms were transferred to the wells	MIC and MBC by microdilution broth assay	• nHAp/Es and nHAp/Sb had antibacterial and antifungal performance against gram-negative bacteria, gram-positive bacteria, and fungi
Rifada et al 46	• 0.25% nHAp • 0.7% nHAp • 1.5% nHAp • Commercial paste (CP) • Distilled water (negative control) • Ampicillin (positive control)	• **Product:** N/A • **Particle size:** N/A	• **Microbial species:** single species of *S. mutans* • **Design:** the biofilm formed in the teeth fragment coated with artificial saliva was treated with slurry toothpaste, which corresponded with the group. Biofilm was allowed to grow for 24 h at 37°C anaerobically	Total count of *S. mutans* bacteria in dental plaque using total plate count method, microenvironment pH using pH strip test	• The presence of nHAp significantly decreased the number of *S. mutans* bacteria present in dental plaque, and the total number of *S. mutans* in the 0.7% nHAp group was equal to the commercial toothpaste samples • nHAp was able to restore salivary pH into neutral within 60 min, with the 0.7% nHAp reaching a pH of 6.7 in 50 min, and commercial paste only returned the pH to 6 within 60 min
El-Said et al 39	• *Jania rubens* red seaweeds nHAp (nHAp-Jr) • *Corallina officinalis* red seaweeds nHAp (nHAp-Co) • Ciprofloxacin	• **Product:** synthesized from *J. rubens* and *C. officinalis* red seaweeds • **Particle size:** 14.62–17.60 nm	• **Microbial species:** single species of *S. mutans* • **Design:** the microdilution technique was employed. The strain was incubated for 24 h at 37°C in BHI and then treated in accordance with the treatment group	Inhibitory percentage, MIC 50, and MIC using XTT assay and cytotoxicity against WISH cell line	• nHAp from both seaweeds showed good efficacy against *S. mutans* compared with ciprofloxacin • Cytotoxicity testing confirmed the relevant antibacterial effects of nHAp-Jr and nHAp-Co without any side effects on hepatocytes
Hariani et al 40	nHAp concentration 12.5, 25, 50, 100, 200 μg/mL	• **Product:** synthesized from snakehead ( *Channa striata* ) fish bones • **Particle size:** 37.32–49.27 nm	• **Microbial species:** single species of *S. aureus* and *E. coli* • **Design:** the test bacteria were each put into a Petri dish containing nutrient agar, then were treated with the test solution and incubated for 24 h at 37°C	Inhibitory diameter using diffusion method	nHAp demonstrated significant antibacterial efficacy against gram-positive *(S. aureus)* and gram-negative *(E. coli)* bacteria
Nambiar et al 34	• 10% nHAp • nHAp and non-collagenous protein (NCP) analogs: 8% sodium tripolyphosphate (STTP) and 3% polyacrylic acid(PAA) • No treatment: negative control • Calcium hydroxide: positive control	• **Product:** commercially purchased from Merck, India • **Particle size:** N/A	• **Microbial species:** *S. mutans* • **Design:** inoculum was spread on the surface of the Mueller–Hinton Agar plate, and then four holes were punched, representing the treatment groups. Each tested paste was introduced into wells. Agar plates were then incubated aerobically for 48 h	Zone of inhibition by agar well diffusion test and number of viable bacteria (CFU) by counting the colony formed	• nHAp had antibacterial efficacy against *S. mutans* . Group 1 (nHAp paste) exhibited the maximum zone of inhibition against *S. mutans* compared with groups 2 and 4. No inhibition zone was observed in group 3 • The last mean value of viable bacteria in group 1 (nHAp paste) indicates better antimicrobial efficacy compared with other groups
Xu et al 47	• nHAp • Zn substituted nHAp • Zn substituted nHAp @polyacrylic acid • nHAp @Alendronate-grafted poly acrylic acid • Zn substituted nHAp @Alendronate-grafte • Phosphate buffered saline treatment (negative control)	• **Product:** N/A • **Particle size:** N/A	• **Microbial species:** single species of *S. mutans* • **Design:** bacterial suspension was co-cultured with nanomaterials for 24 h and inoculated on BHI solid agar plates for 12 h at 37°C	pH of the culture media and inhibition ratio by CFU measurement	nHAp has minimal antibacterial activity against *S. mutans,* and significant improvement was observed in the group of Zn-substituted nHAp
Parajuli et al 41	• 5, 10, 20, 30, 50 mg/mL nHAp • dimethyl sulfoxid (DMSO): negative control	• **Product:** synthesized from waste buffalo bones • **Particle size:** 25 nm	• **Microbial species:** *E. coli* and *S. aureus* • **Design:** the organism was cultivated on MHA agar. Wells were created in the MHA using a sterile 5-cm cork borer. The wells were filled with HAp of varying concentrations mixed with DMSO and used as the negative control. After complete diffusion, the plates were incubated at 36.85°C for 24 h	Zone of inhibition by agar well diffusion method	nHAp has minimal antibacterial activity against *E. coli* and *S. aureu* s
Babayevska et al 42	• Unwaxed dental floss (DF) “fluffy” • Unwaxed DF “smooth” • Waxed DF “smooth” • Nanoparticles prepared by the precipitation method (C1) • Nanoparticles prepared by the hydrothermal method (C2) • nHAp and unwaxed “fluffy” DF (S1) with nHAp, C1 • nHAp and unwaxed “smooth” DF (S2) with nHAp, C1 • nHAp and waxed “smooth” DF (S3) with nHAp, C1 • nHAp and unwaxed “fluffy” DF (S1) with nHAp, C2 • nHAp and unwaxed “smooth” DF (S2) with nHAp, C2 • nHAp and waxed “smooth” DF with (S3) nHAp, C2 • 1 mg/mL rifampicin: positive control	• **Product:** synthesized using a combination of precipitation (directly mixing of Ca ^2+^ and PO _4_^3−^ precursors) and hydrothermal methods (maintaining the nanoparticles in autoclaves at 200°C for 3 h) • **Particle size:** 30 nm in diameter and 150 nm in length	• **Microbial species:** *S. salivarius* • **Design:** *S. salivarius* overnight culture was inoculated into BHI medium test tubes. The test tubes were incubated at 37°C and a speed of 220 rpm after adding materials corresponding to the treatment groups. The microbial growth was evaluated by measuring the optical density at a wavelength of 600 nm at 2-h intervals for the first 6 h, and then at 20, 24, 28, and 44 h following incubation • The paper Whatman disks were dampened with nHAP suspension (containing C1 and C2) and rifampicin (1 mg/mL as positive control). The dehydrated disks, measuring 1 cm in diameter made of DF@HAP, together with pristine dental flosses, were placed into agar plates that had been inoculated with *S. salivarius* (200 μL). The determination of inhibitory zones was determined 24 h after incubation at 37°C. • After a 2-d period of incubation with DF@HAP and pristine dental flosses, *S. salivarius* cells were collected by centrifugation (3 min and 2,000 rpm), rinsed with PBS for 3 min (2,000 rpm), and subsequently stained using fluorescent dyes	Microbial growth based on OD 600-nm measurements, the inhibition zones by diffusion method, and the viability of bacteria population by the fluorescence assay	The application of nHAp-coated with “smooth” dental flosses improved the survival and growth of *S. salivarius* , a probiotic bacterium present in the oral cavity
Ragab et al 35	• nHAp concentration 100, 300, 500, 700, 900 mg/L • Flasks without nanomaterial: negative control	• **Product:** HAp synthesized from a commercial source • **Particle size:** diameter of 19 nm	• **Microbial species:** single species *E. coli* • **Design:** varying concentrations of the tested substance were added to nutrient agar broth medium in Erlenmeyer flasks. Each flask was inoculated with tested bacterium and incubated at 37°C	Cellular respiration, namely, the quantity of O _2_ consumed/min, is conducted using an oxigraph. The flow of K ^+^ was measured using atomic adsorption	• nHAp has an antibacterial effect against *E. coli* • Bacterial growth decreases rapidly and exponentially with increasing nHAp concentration up to a certain concentration of ∼700 mg/L and then slightly decreases
Lamkhao et al 36	• Commercial-HAp • Filtering-HAp using H _2_ O _2_ • Filtering HAp • Microwave-HAp using H _2_ O _2_ • Microwave-HAp	• **Product:** commercial HAp • **Particle size:** N/A	• **Microbial species:** single species *E. coli* and *S. aureus* • **Design:** The samples were positioned on the surfaces of NA and coated with a verified bacterial strain. The NA plates were incubated at 27°C for 24 h	Zone of inhibition (clear zone)	• The antibacterial activity of HAp was not found to be against *E. coli* and *S. aureu* s in the commercial HAp • HAp with antibacterial characteristics can be produced utilizing a microwave-assisted combustion technique
Resmim et al 43	• Negative control for bovine bone • nHAp obtained by calcining bovine bone at 850°C • nHAp obtained by calcining bone at 1,000°C • Negative control for bovine bone • nHAp obtained by calcining porcine bone at 850°C • nHAp obtained by calcining porcine bone at 1,000°C	• **Product:** synthesized from bovine and porcine bones by calcination • **Particle size:** 19–22 nm	• **Microbial species:** single species *S. aureus* • **Design:** a bacterial suspension containing 10 ^5^ CFU/mL was prepared in a saline solution containing NaCl 0.85%. Afterward, the suspension was moved to an Eppendorf tube containing the material and agitated. Subsequently, the combination was evenly spread onto agar plates. The plates were then incubated at 37°C for 24 h	The inhibitory effect by counting the CFU grown on test plates	The natural nHAp derived from the calcining bovine and porcine bones has the ability to inhibit the proliferation of *S. aureus* bacteria. Specifically, the material produced by calcining bovine bones at a temperature of 1,000°C exhibits a superior antibacterial effect against *S. aureus* compared with other substances
Algamal et al 44	• nHAp with a 3% concentration of DMSO in water • nHAp with a 5% concentration of DMSO in water • nHAp with a 7% concentration of DMSO in water • nHAp with a 9% concentration of DMSO in water • nHAp with 100% concentration of DMSO in water	• **Product:** synthesized from marble waste • **Particle size:** N/A	• **Microbial species:** single species *S. aureus* and *E. coli* • **Design:** various test tubes were used to combine varying quantities of water and related solvent (DMSO) with a concentration ranging from 1 to 100% v/v. The incubation was for 24 to 48 h at 35°C	Diameter of the inhibition zone using a well-diffusing method	The nHAp derived from the marble wastes exhibits antibacterial properties against either *E. coli* (gram-negative) or *S. aureus* (gram-positive) bacterial strains
Ibrahim et al 45	• Negative control (without material) • 30% nHAp • 20% nHAp • 10% nHAp	• **Product:** microwaved-synthesized nHAp • **Particle size:** 23.59 nm	• **Microbial species:** single species *S. mutans* and *S. sobrinus* • **Design:** The nHAp powder was added into the 24-well plate with the bacterial suspension and then were incubated in an anaerobic environment at 37°C for 24 h. A serial dilution was performed and plated on BHI agar. The agar plates were incubated anaerobically at 37°C, and the colonies were counted the following day	The percentage of bacterial growth reduction	The application of 30% nHAp significantly reduces the colony counts of both *S. mutans* and *S. sobrinus* compared with the control

### Effect of Hydroxyapatite/Nano-Hydroxyapatite on Bacteria and Fungi

The quantitative measures included in this systematic review to assess the efficacy of nHAp as an antibacterial agent included cell respiration, inhibition capacity (zone, ratio, and percentage), bacterial viability, microbial growth, minimum inhibitory concentration (MIC), mean colony count (MBC), and total count of bacteria. Bacterial adhesion was investigated. The incubation time for antimicrobials in this systematic review was 12 to 48 hours. Eleven studies tested the effectiveness of nHAp as an antibacterial agent against pathogen bacteria only, 32 33 34 35 36 39 40 41 43 44 45 and two studies tested the effectiveness of nHAp as an antibacterial and antifungal agent. 37 38 Out of the 13 studies assessed, 11 studies (85%) demonstrated that nHAp has both antiadhesive and antimicrobial properties against oral pathogenic microorganisms, including gram-negative and gram-positive bacteria, and fungi. 32 34 35 37 38 39 40 43 44 45 46 One study tested the effectiveness of nHAp as an antibacterial agent against nonpathogen bacteria. This study showed that nHAp did not have antimicrobial capabilities against oral nonpathogenic microbes. 42

Five studies compared the antibacterial effects of nHAp against gram-negative ( *E. coli* ) and gram-positive ( *S. aureus* ) bacteria. Two studies showed that nHAp was more effective against gram-negative ( *E. coli* ) 38 41 bacteria, and two studies showed that nHAp was more effective against gram-positive ( *S. aureus* ) bacteria. 37 40 One study did not show any difference in the effectiveness of nHAp against gram-negative and gram-positive bacteria. 44

### Effect of Hydroxyapatite/Hydroxyapatite Nanoparticles on Biofilm

The effectiveness of nHAp as an antibiofilm agent was assessed using several parameters, including biofilm formation, lactic acid generation, biofilm viable count, bacterial count in biofilm, biofilm thickness, metabolic activity, and biomass. The antibiofilm capability of nHAp is heterogeneous.

Seven studies analyzed the effectiveness of nHAp as an antibiofilm. Five studies (71%) showed that HAp and nHAp did not have antibiofilm capabilities against single-species, multispecies, or cross-kingdom biofilms. 30 31 32 33 48 Among these five studies, two conducted antibacterial and antibiofilm tests, and the results showed that nHAp did not have antibacterial and antibiofilm capabilities. 32 33 The other three studies did not carry out antimicrobial tests, so it remains uncertain whether the nHAp in these tests had not only antibiofilm capabilities but also both antimicrobial and antibiofilm capabilities. 30 31 48

Park et al demonstrated enhanced biofilm activity in the group subjected to nHAp small particle size (SPS) treatment. 33 A study by Luo et al also showed increased metabolic activity and lactic acid production of cross-kingdom species in the group treated with nHAp. However, no significant changes were found in metabolic activity and lactic acid production of single-species biofilm. 31

However, two studies (29%) showed that nHAp has antibiofilm capabilities against single-species biofilms. Ahmed et al 37 conducted antimicrobial and antibiofilm tests, and the results showed that nHAp has antibacterial and antibiofilm capabilities, while Rifada et al exclusively conducted antibiofilm tests. 46

### Risk of Bias

The results of the quality assessment of the studies are reported in Table 4 . According to Table 4 , ten studies had a medium risk of bias. 34 35 36 39 40 41 43 44 46 47 Nine other studies had a low risk of bias. 30 31 32 33 37 38 42 45 48 Not all studies described operator details and blinding methods, and seven studies did not employ statistical analysis to examine the findings. 35 36 40 41 43 44 46

**Table 4:** Risk of bias (RoB) of the effect of hydroxyapatite/hydroxyapatite nanoparticles (HAp/nHAp) as antimicrobial and antibiofilm

Study	Clearly stated aims/objectives	Detailed explanation of sample size calculation	Detailed explanation of sampling technique	Details of comparison group	Detailed explanation of methodology	Operator details	Randomization	Method of measurement of outcome	Outcome assessor details	Blinding	Statistical analysis	Presentation of result	Total score	Final score (%)
Luo et al 31	2	1	2	2	2	0	0	2	N/A	N/A	2	2	15	75 ^a^
Huang et al 30	2	1	2	2	2	0	0	2	N/A	N/A	2	2	15	75 ^a^
El-Gar et al 32	2	1	2	2	2	0	0	2	N/A	N/A	2	2	15	75 ^a^
Park et al 33	2	1	2	2	2	0	0	2	N/A	N/A	2	2	15	75 ^a^
Zhang et al 48	2	1	2	2	2	0	0	2	N/A	N/A	2	2	15	75 ^a^
Ahmed et al 37	2	1	2	2	2	0	0	2	N/A	N/A	2	2	15	75 ^a^
Mousavi et al 38	2	1	2	2	2	0	0	2	N/A	N/A	2	2	15	75 ^a^
Rifada et al 46	2	0	2	2	2	0	0	2	N/A	N/A	0	2	12	60 ^b^
El-Said et al 39	2	0	2	2	2	0	0	2	N/A	N/A	2	2	14	70 ^b^
Hariani et al 40	2	0	1	2	2	0	0	2	N/A	N/A	0	2	11	55 ^b^
Nambiar et al 34	2	0	1	2	2	0	0	2	N/A	N/A	2	2	13	65 ^b^
Xu et al 47	2	0	2	2	2	0	0	2	N/A	N/A	2	2	14	70 ^b^
Parajuli et al 41	2	0	1	2	2	0	0	2	N/A	N/A	0	2	11	55 ^b^
Babayevska et al 42	2	1	2	2	2	0	0	2	N/A	N/A	2	2	15	75 ^a^
Ragab et al 35	2	1	1	2	2	0	0	2	N/A	N/A	0	2	12	60 ^b^
Lamkhao et al 36	2	0	1	2	2	0	0	2	N/A	N/A	0	2	11	55 ^b^
Resmim et al 43	2	0	1	2	2	0	0	2	N/A	N/A	0	2	11	55 ^b^
Algamal et al 44	2	0	0	2	2	0	0	2	N/A	N/A	0	2	10	50 ^b^
Ibrahim et al 45	2	1	2	2	2	0	0	2	N/A	N/A	2	2	15	75 ^a^

Criteria: adequately specified = 2 points; inadequately specified = 1 point; not specified = 0 point; and not applicable = exclude criteria from calculus.

*Risk of bias grade judgment was based on the QUIN tool by Sheth et al. 23

Final score (%) = (total score*100)/(2*number of criteria applicable). Risk of bias categories : a = low (final score >70%); b = medium (final score 50–70%); c = high (final score <50%).

## Discussion

Examining the raw material is crucial to determining its effectiveness and efficiency. This is because oral care formulations containing acidic pH values (such as amine fluoride) and other active and inactive ingredients, like ethanol (typically used in conjunction with essential oils), surfactants, preservatives, and others, may affect biofilm management outcomes. 4 49

HAp, a calcium phosphate ceramic, is commonly utilized in biomedical applications owing to its biocompatibility and bioactive properties. The individual oxygen atoms in HAp exist as OH ^−^ ions with hydrogen positioned externally to the cell. Calcium ions are bound by phosphate and hydroxide ions in a coordinated manner. 50 51

This systematic review analyzed the influence of pure HAp in micro and nano size against microbial and biofilm, which plays a role in the occurrence of caries. Compared with standard HAp, nHAp exhibits remarkable features such as a larger surface area, and the ability to penetrate biofilms and damage bacterial cells more effectively. 18 Its small size provides a bigger response surface and superior bioactivity compared with larger crystals. 52 The mechanism of action of nanoparticles involves direct interaction with the bacterial cell wall, eliminating the necessity for cellular penetration; this suggests that nanoparticles may be less likely to induce resistance in bacteria compared with antibiotics. 14

A lot of the research in this systematic review used *S. mutans* as the experimental model to evaluate the antibacterial and antibiofilm effects of HAp/nHAp. It has been suggested that *S. mutans* is the leading cause of human dental caries. The crucial characteristic of the bacterium is its capacity to create a biofilm, referred to as dental plaque, on the tooth surface. 53 Together with glucosyltransferases and other glucan-binding proteins, this organism also synthesizes collagen-binding proteins, protein antigen C, and other substances that work together to form dental plaque and cause dental caries. 54

Dental plaque comprises over 700 distinct bacterial species and has a complex composition. 4 Apart from *S. mutans* , other oral microorganisms that play a role in the occurrence of caries and were analyzed in this study include *S. sobrinus* , *S. gordonii* , *S. aureus* , *E. coli* , *E. faecalis* , and *C. albicans* . *Streptococcus sobrinus* is a predominant oral bacterium associated with developing dental caries. 55 There is a high correlation between the presence of *S. sobrinus* and high caries experience. Children who carry both *S. mutans* and *S. sobrinus* exhibit greater caries experience, caries incidence, and total counts of *S. mutans* compared with those carrying solely *S. mutans* . 56 57

*Streptococcus gordonii* is an initial colonizer of dental plaque. Cells of *S. gordonii* adhere to the tooth surface, initiating a signal transduction pathway termed BrfAB, which modulates adhesive activity. 7 8 *Candida albicans* co-aggregates with *S. gordonii* , *S. sanguinis* , and other α and nonhemolytic *Streptococcus* species, hence stimulating biofilm formation. 58 *Candida albicans* cells are often seen in conjunction with *S. mutans* infection in plaque biofilms from children affected by early childhood caries (ECC). The presence of *C. albicans* in *S. mutans* biofilms can augment the capacity of both species to metabolize sucrose, hence improving the fitness of both organisms within the biofilm community. 7 59 *Staphylococcus aureus* is not a primary driver of caries, but it can potentially influence biofilm resilience and pathology. 60 *Staphylococcus aureus* , *S. mutans* , and *E. coli* were identified from dental caries cases, with *S. aureus* and *S. mutans* exhibiting the highest prevalence. Nonetheless, certain surveys emphasized the involvement of *E. coli* in the development of dental caries, dental plaques, and other associated oral diseases. 61 *Enterococcus faecalis* was predominantly found in the root canals, indicating a potential etiological role in the progression of these diseases. 62 Zhou et al identified *S. mutans* and *C. albicans* as the primary pathogenic microorganisms in irreversible pulpitis in children, whereas *E. faecalis* , *E. coli* , and *S. aureus* are the predominant pathogens in irreversible pulpitis and pulp necrosis in this population. 63

Babayevska et al investigated nonpathogenic bacteria, specifically *S. salivarius* , that contribute to preventing dental caries. *Streptococcus salivarius* may prevent the growth and glucosyltransferase production of *S. mutans* , thereby inhibiting the formation of cariogenic biofilm. This demonstrates that nHAp is safe against nonpathogenic bacteria, which maintain the equilibrium of the oral microbiome. To enhance their protective role, oral care products should be safe from strains of beneficial oral bacteria. 42 64

A common characteristic of many biofilm-controlling agents found in mouthwashes and toothpaste is their ability to eradicate both beneficial and harmful bacteria. 49 Therefore, the primary goal of biofilm management is to maintain a homeostatic state of the oral microbiome. This suggests that using antimicrobials may result in the selection of potentially harmful bacteria and microbiome dysbiosis. 65 66 nHAp may support the growth of probiotic bacteria and act as a mineral reservoir for saliva. The establishment of *S. salivarius* K12 in the oral cavity has the potential to hinder or limit the proliferation of pathogenic bacteria. 67 68

**Fig. 2:**
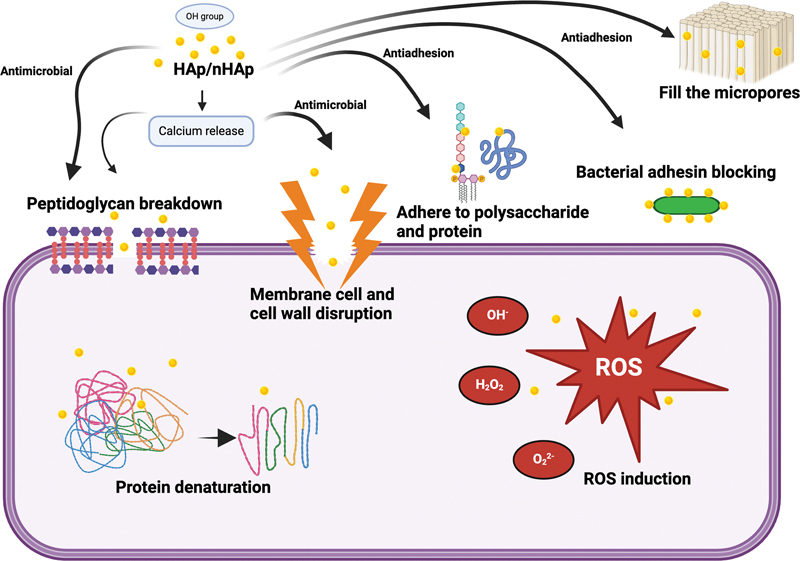
Schematic mechanism of antimicrobial and antibiofilm of hydroxyapatite (Hap)/nano-hydroxyapatite (nHAp) in preventing dental caries.

This systematic review demonstrates the antibacterial capabilities of HAp and nHAp against *S. mutans* , *S. sobrinus* , *S. aureus* , *E. coli* , and *E. faecalis* , antifungal efficacy against *C. albicans* , and the antiadhesion effect against *S. mutans* ( Fig. 2 ). To the best of the authors' understanding, there are no other comprehensive studies examining the antibacterial properties of pure HAp/nHAp. The antibacterial activities of nHAp are attributed to the presence of calcium and other components, which can disturb the bacterial cell wall. 69 In addition to the nanoparticle size, smaller particle sizes, as opposed to larger grain sizes, may enhance antibacterial activity by facilitating quicker ion release. 70 The generation of highly reactive oxygen species (OH ^−^ , H _2_ O _2_ , and O _2_^2−^ ) on the surface of the HAp/nHAp could be one of the possible explanations for the antibacterial efficacy of HAp/nHAp. The primary process that leads to the antibacterial activity is the formation of H _2_ O _2_ , which occurs when H _2_ O _2_ penetrates cell walls. 40

By incorporating N-acetyl muramic acid into mucopeptide structures, the OH group effectively eliminated the bacterial cell wall on the nHAp. The OH group on nHAp denatures the bacterial protein content. 40 The OH group on nHAp causes denaturation of the bacterial protein content. The complex tetrapeptide interactions between the oxygen and hydrogen atoms in HAp and peptidoglycan facilitate the peptidoglycan breakdown. 71 Surface flaws and aggregates contribute to the antibacterial activity of HAp by forming an abrasive surface texture. These aggregates and flaws cause physical damage to the bacteria's cell membrane. 43 Cieplik et al conducted a study in 2020 that revealed the release of calcium ions by synthetic particulate HAp in response to bacterially induced acid challenge within high-concentration *S. mutans* biofilms. The study revealed that HAp displayed a modest buffering capacity within biofilms, increasing to approximately 0.5 pH units. Both Ca ^2+^ and PO _4_^3−^ have buffering effects. The following is a condensed chemical equation that represents acid buffering 72 :

Ca _5_ (PO _4_ ) _3_ (OH) + 7 H ^+^ → 5 Ca ^2+^ + 3 H _2_ PO _4_^−^ + H _2_ O.

Phosphate ions will be released along with calcium ions. Numerous studies have demonstrated that the adhesion of HAp to the tooth surface and its integration into biofilms are crucial factors for achieving a buffering effect in cariogenic biofilms. 73 74 75 76

The effectiveness of HAp/nHAp against gram-positive and gram-negative bacteria cannot be definitively determined due to conflicting results from multiple studies. Two studies indicate higher effectiveness against gram-positive bacteria, while two suggest higher effectiveness against gram-negative bacteria. Additionally, one study found no significant difference in effectiveness between gram-positive and gram-negative bacteria. The observed phenomenon can be attributed to the antibacterial properties of HAp/nHAp, which are influenced by factors such as size, surface area, morphology, porosity, crystallinity, stoichiometry, and the types and concentrations of ions present in the material. 43 77 78

El-Gar et al investigated the antiadhesion ability of nHAp against *S. mutans* . 32 The results of this study showed that nHAp-SPS does not have antiadhesion ability, but HAp modified into nHAp large particle size (LPS) shows antiadhesion ability. This antiadhesion ability of nHAp is in line with the results of a previous review of *in situ* study by Pawinska et al. 79 The mechanism of antiadhesion capabilities is that nHAp could block bacterial adhesins, decrease bacterial adherence, and inhibit bacterial biofilm formation. 78 Proteins and polysaccharides adhering to the HAp's surface prevent bacterial adherence and the formation of biofilms. 80 HAp can also serve as a filler by filling the micropores and indentations on the surface of the enamel. This process forms a new layer of synthetic enamel around the teeth rather than strengthening the current enamel layer. The chemical transformation occurs by converting the enamel into calcium halophosphate (Ca _5_ (PO _4_ )). 32 81 82

Most studies (71%) showed that pure HAp/nHAp did not have antibiofilm capabilities. The analysis was performed on single-species biofilms of *S. mutans* , *S. aureus* , *E. coli* , and *C. albicans* and dual-species biofilms of *S. mutans* and *S. gordonii* , *S. mutans* , and *C. albicans.* Most likely, this is due to the dispersion of the bacteria that form the biofilm community in dental plaque, which exist as separate microcolonies in various physiological settings. The same cells grown in suspended culture display distinct characteristics from those found in biofilm cells. 8 Cariogenic biofilms limit and isolate the chemical's ability to permeate the matrix. As a result, bacteria in biofilms pose a greater challenge for control compared with bacteria in the planktonic phase. They are more resistant to different antibiotics, and the MIC required to combat biofilm bacteria is notably higher (up to 1,000 times) than those needed for planktonic bacteria in a liquid environment. 83 84 85 This outcome is in line with the previous review, which showed that pure nHAp promote the formation of *S. mutans* biofilm by enhancing glucosyltransferase transcription, leading to an increased production of insoluble glucans. 20

Several studies demonstrated that the effectiveness of nHAp as an antibacterial and antibiofilm agent may be possibly enhanced through modification of nHAp or by its combination with other components. The effectiveness of nHAp as an antimicrobial and antibiofilm agent is enhanced by several methods: synthesis using the microwave-assisted combustion method, 36 raising the calcination temperature, 43 disaggregating with sodium hexametaphosphate (SHMP) and ultrasonic cavitation to prevent agglomeration, 30 31 doping with Zn nanoparticles, 47 and dissolving it in DMSO directly. 44

The QUIN RoB tool was used to assess the risk of bias in the selected studies. The instruments consider 12 factors to assess the *in vitro* study's risk of bias. The overall evaluation of bias for all groups produced satisfactory findings, confirming the validity and reliability of the examined *in vitro* research as an essential information source. Table 4 contains visual representations that were included to improve the clarity of the risk of bias findings.

A different recent systematic review has been published regarding the antibacterial and antibiofilm properties of HAp. However, it differs significantly from the current review. The present systematic review exclusively concentrated on *in vitro* experimental investigations, while the systematic review conducted by Limeback et al 86 encompassed clinical trials including human subjects. Limeback et al provide evidence that HAp has anticaries properties by promoting remineralization and decreasing plaque bacterial adherence. 86 Reviews of HAp's antibacterial and antibiofilm properties were also published in two other journals, but they were not registered and were not systematic reviews. 20 49 Imran et al 20 conducted a review only on three studies, namely Park et al, 33 Xu et al, 47 and Ionescu et al. 87 The study's findings suggest that metal-substituted nHAp may enhance its antibacterial properties. The study by Ghosh et al also reviewed the antimicrobial and antibiofilm capabilities of HAp, but it specifically reviewed doped HAp. 80 Meyer and Enax reviewed HAp as a dental care product *in situ* . Their findings demonstrate that HAp, like chlorhexidine, decreases bacterial adhesion to enamel surfaces without eradicating the bacteria. 49

In summary, this systematic review offers significant information on the effects of HAp/nHAp as antimicrobial and antibiofilm agents in preventing dental caries. Despite the variation in the studies included, the results show positive outcomes in their ability to inhibit bacterial growth; however, this material's antibiofilm activity is not at its most effective. The data presented here provide evidence for the potential usefulness of HAp/nHAp in preventive dental applications, highlighting the significance of further research and investigation in this field.

The strengths of this systematic review encompass the quality of its methodology, guided by the Cochrane Handbook for Systematic Reviews of Interventions, and the review was registered in the OSF. The limitation of this systematic review is that the investigation was only conducted through *in vitro* experiments. Various antibacterial and antibiofilm analysis techniques were employed in the study in conjunction with specific microorganism species. Further systematic reviews are required to conduct *in vivo* and *in situ* studies to precisely ascertain the clinical antibacterial and antibiofilm properties of HAp/nHAp. In addition, because it was challenging to pinpoint the causes of variability, the systematic review was predicated on the idea that antimicrobial and antibiofilm evaluation was uniform among the studies. Consequently, a heterogeneity analysis was not conducted because few comparable studies were available.

## Conclusion

The presented literature review indicates that pure HAp/nHAp has promise as a viable biomimetic substitute for conventional antibacterial agents in oral care. Nevertheless, the effectiveness of this compound in combating biofilm formation is still insufficient. Antibiofilm capabilities can be increased by modifying the synthesis of nHAp or combining nHAp with other materials known to have antibiofilm capabilities. Future studies should prioritize investigating methods to enhance the effectiveness of nHAp in combating biofilm formation.
