# Supplementary material for: Antimicrobial and Antibiofilm Properties of Hydroxyapatite/Nano-Hydroxyapatite in Preventing Dental Caries: A Systematic Review
Source: Eur J Dent. 2025 May 1;19(3):563–79. doi: 10.1055/s-0045-1802568 (PMC12182413; doi:10.1055/s-0045-1802568)
Supplement: Supplementary file 1 — Supplementary Material [file 10-1055-s-0045-1802568-s2493801.pdf]

Supplementary Material S1 PRISMA 2020 for abstracts checklist

Section and topic	Item no.	Checklist item	Reported (yes/no)
**Title**			
Title	1	Identify the report as a systematic review	Yes
**Background**			
Objectives	2	Provide an explicit statement of the main objective (s) or question(s) the review addresses	Yes
**Methods**			
Eligibility criteria	3	Specify the inclusion and exclusion criteria for the review	Yes
Information sources	4	Specify the information sources (e.g., databases, registers) used to identify studies and the date when each was last searched	Yes
Risk of bias	5	Specify the methods used to assess risk of bias in the included studies	Yes
Synthesis of results	6	Specify the methods used to present and synthesis results	Yes
**Results**			
Included studies	7	Give the total number of included studies and participants and summaries relevant characteristics of studies	Yes
Synthesis of results	8	Present results for main outcomes, preferably indicating the number of included studies and participants for each. If meta-analysis was done, report the summary estimate and confidence/credible interval. If comparing groups, indicate the direction of the effect (i.e., which group is favored)	Yes
**Discussion**			
Limitations of evidence	9	Provide a brief summary of the limitations of the evidence included in the review (e.g., study risk of bias, inconsistency, and imprecision)	Yes
Interpretation	10	Provide a general interpretation of the results and important implications	Yes
**Other**			
Funding	11	Specify the primary source of funding for the review	No
Registration	12	Provide the register name and registration number	No

Source: Page et al.[88]
Note: For more information, visit http://www.prisma-statement.org/.

Supplementary Material S2 PRISMA 2020 checklist

Section and topic	Item no.	Checklist item	Location where item is reported
**Title**			
Title	1	Identify the report as a systematic review	Title page
**Abstract**			
Abstract	2	See the PRISMA 2020 for abstracts checklist	Lines 1–19
**Introduction**			
Rationale	3	Describe the rationale for the review in the context of existing knowledge	Lines 61–65
Objectives	4	Provide an explicit statement of the objective(s) or question (s) the review addresses	Lines 65–68

 © 2025. The Author(s).

Supplementary Material S2 (*Continued*)

Section and topic	Item no.	Checklist item	Location where item is reported
**Methods**			
Eligibility criteria	5	Specify the inclusion and exclusion criteria for the review and how studies were grouped for the syntheses	Lines 92–100<br>Table 2
Information sources	6	Specify all databases, registers, Web sites, organizations, reference lists, and other sources searched or consulted to identify studies. Specify the date when each source was last searched or consulted	Lines 81–83
Search strategy	7	Present the full search strategies for all databases, registers, and Web sites, including any filters and limits used	Lines 83–88<br>Table 1
Selection process	8	Specify the methods used to decide whether a study met the inclusion criteria of the review, including how many reviewers screened each record and each report retrieved, whether they worked independently, and, if applicable, details of automation tools used in the process	Lines 101–107
Data collection process	9	Specify the methods used to collect data from reports, including how many reviewers collected data from each report, whether they worked independently, any processes for obtaining or confirming data from study investigators, and, if applicable, details of automation tools used in the process	Lines 101–107<br>Suppl. material
Data items	10a	List and define all outcomes for which data were sought. Specify whether all results that were compatible with each outcome domain in each study were sought (e.g., for all measures, time points, analyses), and if not, the methods used to decide which results to collect	–
	10b	List and define all other variables for which data were sought (e.g., participant and intervention characteristics, funding sources). Describe any assumptions made about any missing or unclear information	–
Study risk of bias assessment	11	Specify the methods used to assess risk of bias in the included studies, including details of the tool(s) used, how many reviewers assessed each study and whether they worked independently, and, if applicable, details of automation tools used in the process	Lines 122–127
Effect measures	12	Specify for each outcome the effect measure(s) (e.g., risk ratio, mean difference) used in the synthesis or presentation of results	–
Synthesis methods	13a	Describe the processes used to decide which studies were eligible for each synthesis (e.g., tabulating the study intervention characteristics and comparing against the planned groups for each synthesis [item no. 5])	Lines 109–119
	13b	Describe any methods required to prepare the data for presentation or synthesis, such as handling of missing summary statistics, or data conversions	Lines 110–112
	13c	Describe any methods used to tabulate or visually display results of individual studies and syntheses	Lines 112–117
	13d	Describe any methods used to synthesize results and provide a rationale for the choice(s). If meta-analysis was performed, describe the model(s), method(s) to identify the presence, and extent of statistical heterogeneity, and software package(s) used	Lines 117–119
	13e	Describe any methods used to explore possible causes of heterogeneity among study results (e.g., subgroup analysis, meta-regression)	–
	13f	Describe any sensitivity analyses conducted to assess robustness of the synthesized results	–

(*Continued*)

 © 2025. The Author(s).

Supplementary Material S2 (*Continued*)

Section and topic	Item no.	Checklist item	Location where item is reported
Reporting bias assessment	14	Describe any methods used to assess risk of bias due to missing results in a synthesis (arising from reporting biases)	Lines 122–127
Certainty assessment	15	Describe any methods used to assess certainty (or confidence) in the body of evidence for an outcome	–

PRISMA 2020 checklist

Section and topic	Item no.	Checklist item	Location where item is reported
**Results**			
Study selection	16a	Describe the results of the search and selection process, from the number of records identified in the search to the number of studies included in the review, ideally using a flow diagram	Lines 130–138 Fig. 1
	16b	Cite studies that might appear to meet the inclusion criteria, but which were excluded, and explain why they were excluded	Lines 134–138
Study characteristics	17	Cite each included study and present its characteristics	Lines 139–154 Table 1
Risk of bias in studies	18	Present assessments of risk of bias for each included study	Lines 202–207 Table 4
Results of individual studies	19	For all outcomes, present, for each study: (a) summary statistics for each group (where appropriate) and (b) an effect estimates and its precision (e.g., confidence/credible interval), ideally using structured tables or plots	–
Results of syntheses	20a	For each synthesis, briefly summarize the characteristics and risk of bias among contributing studies	Lines 161–207 Table 3
	20b	Present results of all statistical syntheses conducted. If meta-analysis was done, present for each the summary estimate and its precision (e.g., confidence/credible interval) and measures of statistical heterogeneity. If comparing groups, describe the direction of the effect	–
	20c	Present results of all investigations of possible causes of heterogeneity among study results	Lines 139–154
	20d	Present results of all sensitivity analyses conducted to assess the robustness of the synthesized results	–
Reporting biases	21	Present assessments of risk of bias due to missing results (arising from reporting biases) for each synthesis assessed	Lines 205–207
Certainty of evidence	22	Present assessments of certainty (or confidence) in the body of evidence for each outcome assessed	–
**Discussion**			
Discussion	23a	Provide a general interpretation of the results in the context of other evidence	Lines 310–330
	23b	Discuss any limitations of the evidence included in the review	Lines 331–335
	23c	Discuss any limitations of the review processes used	Lines 335–338
	23d	Discuss implications of the results for practice, policy, and future research	Lines 341–346

 © 2025. The Author(s).

(*Continued*)

Section and topic	Item no.	Checklist item	Location where item is reported
**Other information**			
Registration and protocol	24a	Provide registration information for the review, including register name and registration number, or state that the review was not registered	Lines 72–74
	24b	Indicate where the review protocol can be accessed, or state that a protocol was not prepared	Lines 73–74
	24c	Describe and explain any amendments to information provided at registration or in the protocol	–
Support	25	Describe sources of financial or nonfinancial support for the review, and the role of the funders or sponsors in the review	Line 349
Competing interests	26	Declare any competing interests of review authors	Line 352
Availability of data, code, and other materials	27	Report which of the following are publicly available and where they can be found: template data collection forms; data extracted from included studies; data used for all analyses; analytic code; any other materials used in the review	Suppl. material

Source: Page et al.[88]
Note: For more information, visit http://www.prisma-statement.org/.

Supplementary Material S3 Screener instructions

Antimicrobial and Antibiofilm Properties of Hydroxyapatite/Hydroxyapatite-Nanoparticles in Preventing Dental Caries: A Systematic Review

Title/Abstract

Objective
Critically investigate and map the evidence on the antimicrobial and antibiofilm properties of HAP/HAP-NPs in preventing dental caries by mapping the existing literature.

Current review phase
This first phase of the review will include screening the title and abstract of each article that is generated through our search to assess their eligibility for inclusion in this review. After reading the title and abstract of the article, please mark the article as "included" if it meets *all* of the inclusion criteria or as "excluded" if meets *one or more* of the exclusion criteria. If you are unsure if the article meets the criteria, please *include* the article.

The following criteria should be assessed:

Construct	Inclusion criteria *(include if article meets all)*	Exclusion criteria *(exclude if article meets one)*
Language	Article published in English	Article is published in a language other than English
Publication	Peer-reviewed articles with association full papers	Abstracts without the associated full papers, studies in case reports, conference papers, book chapters, patents, letters to the editor, and studies not reporting statistical data
Data	Primary data collection and analysis	Systematic reviews, meta-analysis, and literature review papers
Context		

(*Continued*)

 © 2025. The Author(s).

(*Continued*)

Construct	Inclusion criteria *(include if article meets all)*	Exclusion criteria *(exclude if article meets one)*
	All species of oral bacteria and oral candida that cause dental caries	Any other oral bacteria and oral candida that not cause dental caries; oral viruses
Intervention	Addition of hydroxyapatite/hydroxyapatite nanoparticles	Additional active ingredients other than hydroxyapatite
Intervention outcomes	Progression or regression of cariogenic microbial or biofilm (progression **or** regression)	No relevant outcome reported
Study designs	*In vitro* and *in vivo* studies	Other preclinical research and clinical research

Full Text

This phase of the review will include reading the full-text of studies that have been accepted in the last screening phase to determine if they are eligible for inclusion in this review. While reading the article, if it meets *one or more* exclusion criteria, please mark the article as "excluded" and select the corresponding reason from the drop-down menu. Upon completing the article, if the article meets *all* of the inclusion criteria, please mark the article as "included."

Construct	Inclusion criteria *(include if article meets all)*	Exclusion criteria (*exclude if article meets one)*
Language	Article published in English	Article is published in a language other than English<br>**Covidence label:** Article not written in English
Publication	Peer-reviewed articles with association full papers	Abstracts without the associated full papers, studies in case reports, conference papers, book chapters, patents, and letters to the editor<br>**Covidence label:** Article not peer reviewed with association full papers and reporting statistical data
Data	Primary data collection and analysis	Systematic reviews, meta-analysis, and literature review papers<br>**Covidence label:** Not a primary data collection or analysis
Context	All species of oral bacteria and oral candida that cause dental caries	Any other oral bacteria and oral candida that do not cause dental caries; oral viruses<br>**Covidence label:** Participants do not cause dental caries
Implementation	Addition of hydroxyapatite/hydroxyapatite nanoparticles	Additional active ingredients other than hydroxyapatite<br>**Covidence label:** Intervention not addition of hydroxyapatite/hydroxyapatite nanoparticles only
Goal	Progression or regression of cariogenic microbial or biofilm	No relevant outcome reported<br>**Covidence label:** No relevant outcome reported
Study designs	*In vitro* and *in vivo* studies	Other preclinical research and clinical research<br>**Covidence label:** Study designs not *in vitro* or *in vivo*

 © 2025. The Author(s).

Supplementary Material S4 Extractor instructions

Antimicrobial and Antibiofilm Properties of Hydroxyapatite/Hydroxyapatite-Nanoparticles in Preventing Dental Caries: A Systematic Review

Please read through each article assigned to you and complete the extraction table with the relevant information. When possible, please use direct quotes from the article to answer each question. When using direct quotes, please mark the quote with quotation marks (“”) and start new quotes on a new line. When it is not possible to use direct quotes, summarizing/paraphrasing from the article is acceptable.

If the information for the question is not included in the article, please write “not specified” in the answer fields.

The following extraction questions will be uploaded to Microsoft Excell Spreadsheet where responses will be input:

Article Information

1. Article title
2. Article author
3. Year of publication
4. Study design

Context

5. Studied microbes
6. Studied biofilms

Intervention

7. Preparation form
8. Source of HAp
9. Particle size
10. HAP concentration

Intervention outcomes

11. Assay (s)
12. Statistical analysis
13. Recorded result

 © 2025. The Author(s).
